# Direct Transmission of Digital Message via Programmable Coding Metasurface

**DOI:** 10.34133/2019/2584509

**Published:** 2019-01-16

**Authors:** Tie Jun Cui, Shuo Liu, Guo Dong Bai, Qian Ma

**Affiliations:** ^1^State Key Laboratory of Millimeter Waves, Southeast University, Nanjing 210096, China; ^2^Synergetic Innovation Center of Wireless Communication Technology, Southeast University, Nanjing 210096, China; ^3^Jiangsu Xuantu Technology Co., Ltd., 12 Mozhou East Road, Nanjing 211111, China

## Abstract

In modern wireless communications, digital information is firstly converted to analog signal by a digital-analog convertor, which is then mixed to high-frequency microwave to be transmitted through a series of devices including modulator, mixer, amplifier, filter, and antenna and is finally received by terminals via a reversed process. Although the wireless communication systems have evolved significantly over the past thirty years, the basic architecture has not been challenged. Here, we propose a method to transmit digital information directly via programmable coding metasurface. Since the coding metasurface is composed of ‘0' and ‘1' digital units with opposite phase responses, the digital information can be directly modulated to the metasurface with certain coding sequences and sent to space under the illumination of feeding antenna. The information, being modulated in radiation patterns of the metasurface, can be correctly received by multiple receivers distributed in different locations. This method provides a completely new architecture for wireless communications without using complicated digital-analog convertor and a series of active/passive microwave devices. We build up a prototype to validate the new architecture experimentally, which may find promising applications where information security is highly demanded.

## 1. Introduction

It has been a long time since human begins to communicate over a long distance. The history of telecommunication can date back to more than 5,000 years ago, including the use of smoke signals in China and drums in Africa. These ancient attempts rely on the vision and hearing of human beings, some of which are still in use today, such as sign language by traffic police and semaphore signal by voyage. With the invention of telegram and discovery of electromagnetic (EM) wave in the 19th century, the way people transmit message to far places has experienced revolutionary changes. The messages can be delivered with the speed of light through metal wire, or even air, using electricity or EM waves as the carriers.

Supplementary [Supplementary-material supplementary-material-1] presents an architecture of modern wireless communication system [1, 2]. The information to be sent is firstly converted into binary codes, which can be easily stored and processed by electronic devices. Just like in ancient times when people deliver information with different arm's positions in flag semaphore or with different durations of sound in drum telegraphy, researchers in wireless communications have been focusing on how messages can be efficiently encoded and wirelessly transmitted to terminals with the maximum speed and minimum error. This goal is achieved by digital modulation, one of the most important modules in the communication system, which determines the transmission rate and bit error rate. We briefly review some popular digital modulation techniques in Supplementary [Supplementary-material supplementary-material-1], including the amplitude-shift keying (ASK), frequency-shift keying (FSK), and phase-shift keying (PSK) [3], in which the digital baseband signals are represented by the variations in the amplitude, frequency, and phase of a reference signal (or carrier wave), respectively. A more complicated modulation method, called as quadrature amplitude modulation (QAM) [4], further exploits the physical bandwidth to enhance the transmission rate by using two mutually orthogonal carrier signals. As the frequency of the digital information is too low to be sent directly via radio-frequency (RF) wave, it has to be converted to analog signals using a digital-to-analog converter (DAC) and then is modulated to high-frequency carrier wave via mixers. For the brevity of content, some modules are omitted in the schematic in Supplementary [Supplementary-material supplementary-material-1], such as digital up convertor (DUC), which up-shifts the digitally modulated signal to digital intermediate frequency. The modulated signal containing the coded information is further amplified by a series of power amplifier circuits and is finally radiated to free space through an antenna.

In all existing wireless communication systems, the digital information is modulated by manipulating the amplitude, phase, and frequency of the carrier wave. In the proposed new wireless communication system, however, different digital coding sequences will be directly modulated to the metasurface, resulting in different electric-field distributions in the far-field region (i.e., far-field radiation patterns). Hence it is possible to use the variation of far-field radiation patterns to modulate the digital signal. This interesting finding may fundamentally change the architecture of the modern wireless communication system. Then it comes to a question: can the far-field radiation pattern be used as an extra dimension to transmit digital information to receiver wirelessly? The answer is yes, as illustrated in the last row of Supplementary [Supplementary-material supplementary-material-1], in which the single-beam and dual-beam radiation patterns represent the binary digital codes ‘0' and ‘1, respectively, so that the digital information (e.g., 0101101) can be transmitted to the far fields by dynamically generating such two radiation patterns according to the coding sequence. Based on the recently proposed programmable metamaterials [4], we propose a new wireless communication architecture, which is called as directly digital modulation (DDM).

Before introducing the concepts of direct transmission of digital information and new architecture of wireless communications, we give a brief review to the digital coding and programmable metamaterials [5]. They can be viewed as the digital version of conventional metmaterials [6], which are composed of an array of digital particles, with each state being dynamically controlled by an field programmable gate array (FPGA) at the ‘0' and ‘1' states (i.e., two reflection responses with equal amplitude but opposite phases). By applying pre-designed coding sequences to the programmable metamaterial, both near and far fields of EM waves can be manipulated in real time as desired manner, such as anomalous reflection, beam splitting, and random diffusion [7]. Dynamically changing radiation patterns of the programmable metamaterial offers a new perspective to microwave applications and has led to some exciting systems like reprogrammable metasurface hologram [8] and single-sensor and single-frequency imaging [9]. Recently, the digital coding and programmable metamaterial has experienced rapid development and received wide attention from microwave [8–12] and terahertz wave [7, 13, 14] to acoustic frequency [15, 16]. Some milestone works include Minkowski-fractal-shaped coding metamaterial to make broadband terahertz diffusion [6], anisotropic digital metamaterial to realize dual-functional performance under orthogonal polarizations [12, 13], and tensor digital coding metasurface to produce highly efficient conversion from spatially propagating wave to surface wave [14]. It is important to remark that the digital coding description allows metamaterial and metasurface to be studied by digital signal processing methods to reach more flexible controls of EM waves in much easier processes [10, 17]. The readers may refer to comprehensive reviews on this topics [18, 19], which demonstrate the basic concept, working principle, design method, fabrication, and experimental validation of digital coding and programmable metamaterials and metasurfaces.

## 2. Results

### 2.1. Working Mechanism of the DDM System

Based on the digital coding and programmable metasurface, here we build up an entirely new architecture for the wireless communications. The digital information, expressed in form of binary coding sequence (e.g., 00101001…), is firstly encoded on the metasurface in a programmable way via FPGA, and then radiated to free space directly by the metasurface in the form of EM waves under the illumination of feed antenna. Because the radiated far field is dependent on the digital coding sequence, we use multiple receiving antennas pointing to different directions to obtain the transmitted digital information by reading the radiation pattern. In the new architecture, the complicated digital-analog convertor, modulator, mixer, and a series of other active and passive microwave devices used in the conventional system are not required. More importantly, the digital information can only be correctly recovered from the shape of radiation pattern instead of a single point, which implies that the far field captured at any single direction cannot recover the original digital information correctly. This important feature makes the proposed DDM architecture a naturally secret communication system. Unlike the conventional communication systems where the transmitted information is encrypted by software approach using various encryption algorithms, the digital information sent by the DDM system is fully protected from interception at a single or multiple locations from the physical level, without using the software encryption.

**Figure 1:**
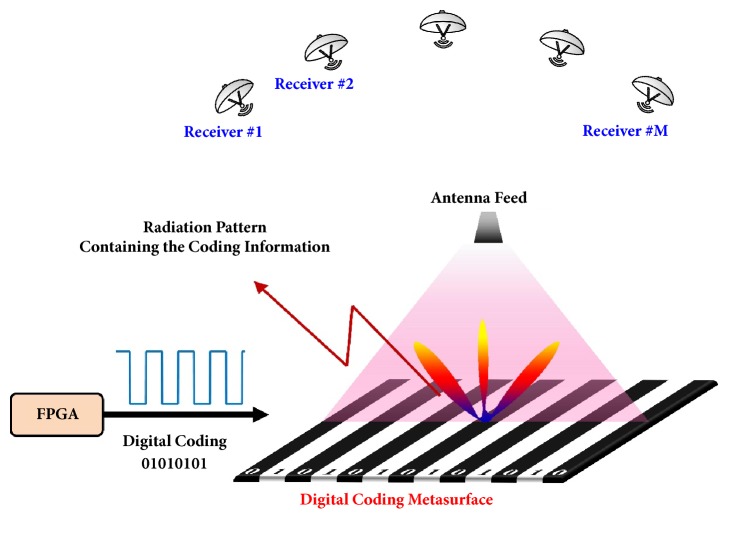
Schematic illustration of the working mechanism of the proposed DDM system.

Figure 1 presents the architecture of DDM system, which only comprises of an FPGA module and a digital coding metasurface. The information stream to be sent out is firstly divided into multiple fragments of* n*-bit binary code (e.g. ‘0101'), which is hereinafter called as information code. Each information code is then mapped to an* m*-bit (*m*>*n*) binary code (called as hardware code, such as ‘01010101') using specially designed channel optimization algorithm, which will be described in later discussions. In each symbol period, the* m*-bit hardware code is amplified to control the digital state of each coding particle on metasurface, which has a number of (*m*) independently controllable columns. Under the quasiplane-wave illumination of feeding antenna, the coding metasurface will radiate the digital coding information directly to free space in the form of specific radiation pattern. The entire digital message can be therefore transmitted with fast changing hardware codes on the programmable metasurface. Because each hardware code determined by the channel optimization algorithm corresponds to a unique radiation pattern, the digital information can be correctly recovered by making one-to-one mapping between the received radiation pattern and hardware code. Usually, a number of receivers are used to capture the radiation pattern, as shown in Figure 1. As a proof of concept for the working mechanism of the DDM system, we consider a simple one-dimensional (1D) digital coding metasurface, in which the digital states of the coding particles are controlled by columns. In the 1D case, only two-dimensional (2D) radiation patterns on elevation plane are required to recover the original digital information. We remark that the same working principle applies to the design of 2D DDM systems, which enable much higher data transmission rates.

To demonstrate the performance of the DDM architecture in terms of its transmission rate and robustness against noise, we take a simple metasurface with* N*=5 columns as the illustrative example, which has totally 2^5^=32 different coding patterns. However, if we only consider their amplitudes, some of the 32 radiation patterns are identical due to the 180° phase difference between ‘0' and ‘1' digital states. In fact, the *n*^th^ coding pattern and the (*5-n*)^th^ coding pattern are equivalent because they have the same radiation pattern. For instance, ‘00000' is equivalent to ‘11111,' and ‘00001' is equivalent to ‘11110.' Thus, we only need to generate the first 16 coding patterns from ‘00000' to ‘01111,' as illustrated in Supplementary [Supplementary-material supplementary-material-1]. Supposing that such coding sequences vary along the* x*-direction and are illuminated by a plane wave, we could calculate the corresponding radiation patterns using a fast method based on Fourier transform [20], as shown in Supplementary [Supplementary-material supplementary-material-1].

**Figure 2:**
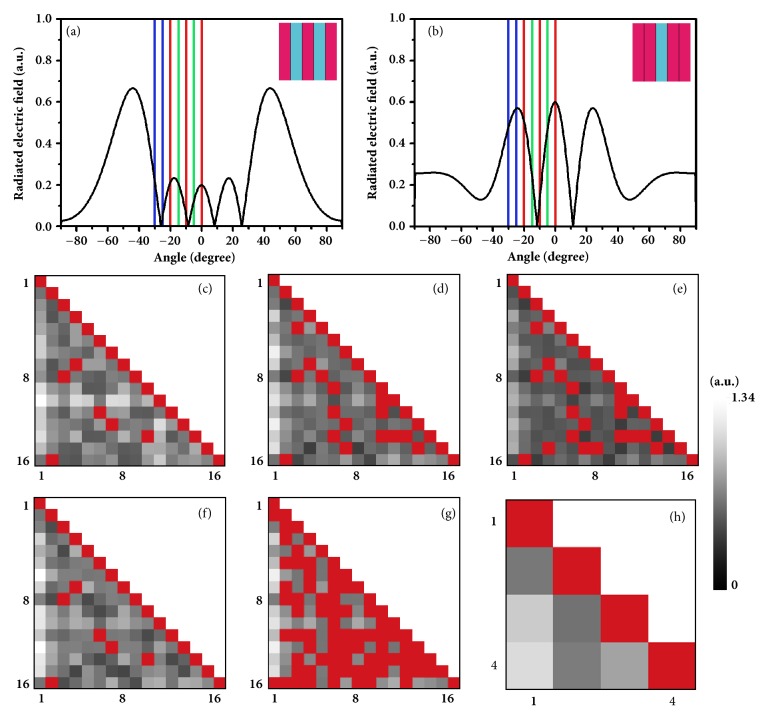
**Demonstration of the working mechanisms of the channel estimation and optimization algorithms.** (a, b) The radiation patterns in the* x-z* plane for the coding patterns with hardware codes 01010 and 00100, respectively. (c-g) The distance matrices for the 16 digital states obtained under different receiving angels and noise thresholds: (c) at all receiving angles and noise threshold 0; (d) at receiving angles 0°, 5°, 10°, 15°, and 20° and noise threshold 0.3; (e) at receiving angles 0°, 10°, and 30° and noise threshold 0.3; (f) at receiving angles 0°, 5°, 10°, 15°, 20°, 25°, and 30° and noise threshold 0.3; (g) at receiving angles 0°, 5°, 10°, 15°, and 20° and noise threshold 0.6. (h) The distance matrix for the 4 available digital states after the optimization treatment to the case in (g).

Figures 2(a) and 2(b) present the radiation patterns of two coding sequences (hardware codes) ‘01010' and ‘00100', respectively. The obvious difference between these radiation patterns allows the receivers to distinguish and recover the transmitted digital information. In order to quantitatively analyze the difference between radiation patterns, we calculate the Euclidean distances between every two of the 16 radiation patterns and display them in a 16×16 distance matrix, as shown in Figure 2(c). The gray level of each pixel (*m*,* n*) indicates the relative distance between *m*^th^ and *n*^th^ radiation patterns; and the red pixels located at (16, 2), (9, 3), (8, 4), (12, 6), (13, 7), and (14, 10) indicate zero distance, making it impossible to distinguish from each other. Please refer to Supplementary Information for detailed discussions on these duplicated digital states. By performing a channel estimation algorithm to the distance matrix, we obtain 10 available digital states (Nos. 1, 2, 3, 4, 5, 6, 7, 10, 11, and 15) that can be used for the unambiguous transmissions. If we select 8 digital states from them, the system can transmit 3 bits per symbol.

However, in real applications, it is unnecessary and impractical to record the entire radiation pattern at every angle from -90° to 90°. The maximum angle (with respect to the normal of the coding metasurface) of the received radiation pattern is determined by real application situations. The larger the maximum receiving angle is, the more information will be captured. However, the path difference between signals passing through the minimum and maximum radiation angles cannot be neglected, especially for large receiving angles (>60°), because such a path difference leads to a non-negligible time delay of signals and may reduce the symbol rate. For this reason, we only sample the radiation pattern at some discrete angles from 0° to 30°, as marked by the colored lines in Figures 2(a) and 2(b). Figure 2(d) shows the distance matrix when the radiation patterns are sampled at five angles of 0°, 5°, 10°, 15°, and 20°, as marked by the red and green lines in Figure 2(a). Since the radiation pattern is symmetric with respect to the 0° angle, only the radiation pattern in the positive angle range is sampled.

Considering the inevitable background noise and limited sensitivity of the receivers, those distances smaller than a certain noise threshold (set as 0.3 here) are considered as indistinguishable (marked by red pixels). As a consequence, the number of available digital states decreases to 6, which are numbers 1, 2, 3, 4, 6, and 7, supporting a 2-bit symbol transmission. If we reduce the number of sampling to only three receiving angles 0°, 10°, and 20° (see the red lines), the average Euclidean distance between the radiation patterns will decrease accordingly, as can be observed in Figure 2(e), in which more pixels are darker than those in Figures 2(c) and 2(d). However, the available digital states in this case still maintain the same as those in Figure 2(d), which means that a smaller sampling resolution does not necessarily lead to fewer available digital states. Instead, increasing the number and angle range of sampling can effectively increase the average Euclidean distance of the distance matrix, which helps improve system robustness against background noise. This feature is verified from the lighter Euclidean distance matrix shown in Figure 2(f), where the sampling angles increase to 0°, 5°, 10°, 15°, 20°, 25°, and 30° (see the blue, red, and green lines).

### 2.2. Noise Effect and Channel Optimization Algorithm

In practical applications, noise is everywhere and cannot be neglected in the design of wireless communication systems. The noise-resistant performance of the system determines its channel quality and transmission rate [21]. Many techniques have been proposed in the past decades to improve the performance of wireless communication systems under high noise environments. One of the effective methods to improve the channel quality is to increase the signal-to-noise ratio (SNR), which can be realized by increasing transmission power, or using multiple-input and multiple-output (MIMO) technology [22]. But such methods cause extra power consumption and increased system complexity, which are not feasible for devices with limited power and space, such as satellites. Therefore, a lot of efforts have been devoted to improve the transmission rate under a given SNR with some capacity approaching codes, such as turbo code [23] and low-density parity-check code (LPDC) [24], allowing the information to be transmitted with a data rate approaching the Shannon limit.

For this reason, it is necessary to characterize the performance of the new architecture under high noise level. Figure 2(g) shows the distance matrix when the radiation pattern is sampled at the same angles as Figure 2(d) but with larger noise threshold of 0.6. As a result, the darker pixels with values smaller than 0.6 in Figure 2(d) now become zero distances (marked by red colors in Figure 2(g)). The ten digital states are now merged as a single state through the complex connections (zero distance) among individual states, resulting in the worst condition where no information can be transmitted.

Thus, we have to ask a question: is it possible to increase the number of available digital states from the software level without increasing SNR of the system, just like what turbo code or LDPC code does in modern communication systems? Fortunately, we succeed in finding a channel optimization algorithm that can effectively increase the number of available digital states by discarding some critical ones. Taking the 2D case as example, in which the radiation pattern is sampled at two different receiving angles and all digital states can be expressed in 2D Cartesian coordinate system, we illustrate in Supplementary Information how the number of available digital states can be increased by deleting some useless states. Performing the optimization algorithm to the distance matrix in Figure 2(g), we observe that the available digital states increase to four (Nos. 1, 3, 4, and 11), which could still support the same 2-bit-symbol rate as that in Figure 2(d). Figure 2(h) provides the corresponding distance matrix of the four selected digital states, from which we find that all mutual distances are larger than zero, demonstrating the effectiveness of the optimization algorithm in the improvement of available digital states without increasing the transmitting power or the sensitivity of receivers.

**Figure 3:**
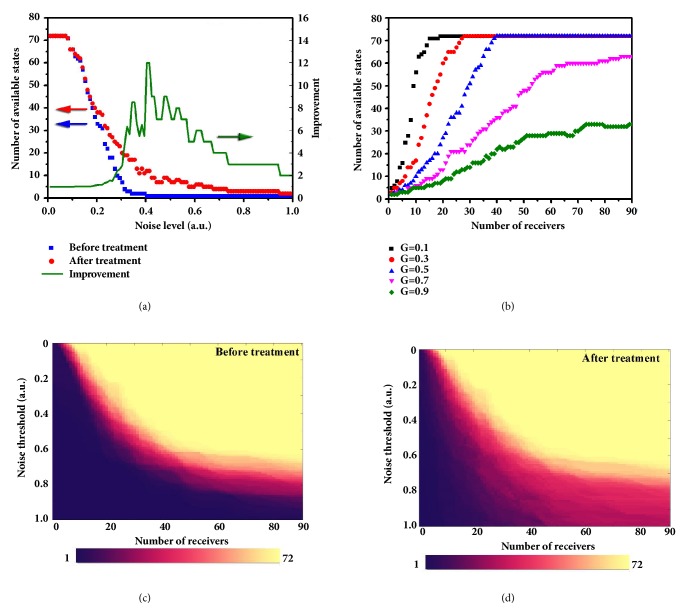
**Quantitative analyses of the channel optimization algorithm under different noise levels and numbers of receivers for a 9-column DDM system.** (a) The variation of numbers of the available digital states before and after the optimization treatment as the noise level increases from 0 to 1. The green line represents the improvement (ratio) of the numbers of available digital states before and after the optimization treatment. (b) The variation of numbers of the available digital states under different noise levels as the number of receivers increases from 1 to 90. Note that the number is counted from 1° with step of 1°. (c, d) The dependences of numbers of the available digital states on the number of receivers and the noise threshold, respectively.

To have a better insight into the performance of the channel optimization algorithm, we present a quantitative analysis on improving the number of available digital states under different noise levels and spatial sampling resolutions. Taking the 9-column DDM system as example, Figure 3(a) shows how the number of available digital states varies as a function of the noise threshold before (blue square symbol) and after (red round symbol) making the channel optimization. Note that the radiation pattern is sampled by five receivers located at 0°, 5°, 10°, 15°, and 20°. It is apparent that both curves experience a rapid drop as the noise level increases from 0.1 to 0.3, indicating that a noise threshold exists. After the threshold, the system performance drops off precipitously. However, with the optimization treatment, the number of available digital states increases by a factor of at least 4 in the noise level range from 0.3 to 0.67 and reaches the maximum of 12 when the noise level equals 0.41, which is equivalent to a 2-3 times enhancements in the transmission rate. One may notice that the number of available digital states decreases to 1 when the noise level exceeds 0.4, at which no information can be transmitted. It is surprising to see that the optimized number of available digital states can support at least a 1-bit-symbol transmission in the entire noise level range (noise level<1) and even transmit 2- and 3-bit symbols if the noise level is not very large (noise level<0.72).

The above example demonstrates remarkable performance of the channel optimization algorithm on the number of available digital states. Now we analyze how the number of receivers affects the available digital states at different noise levels (G), as shown in Figure 3(b). Here, the horizontal axis represents the number of receivers, which starts from 1 with step of 1, and all cases have been treated with the optimization algorithm. As expected, all curves tend to rise with the increase of receivers' number. When the noise level is as low as 0.1, the number of available digital states rises rapidly as the number of receiver increases from 1 to 15. When the number of receiver exceeds the critical point 19, the system reaches the best condition with a maximum number of 72 available digital states. This means that the number of available digital states reaches saturation once the number of receiver is over the critical point. It can be concluded that when the DDM system is in a low noise environment, only a small proportion of the radiation pattern is required to realize the maximum-rate transmission. If we increase the noise threshold to 0.3 and 0.5, the slopes of curves gradually decrease, resulting in a larger critical point of 28 and 40, respectively. However, as the noise threshold is further lifted to 0.7 and 0.9, the system performance deteriorates so seriously that the best condition cannot be achieved even though the radiation pattern is sampled at every angle from 1° to 90°. Fortunately, the number of available digital states under G=0.9 is still enhanced significantly by a maximum factor of 16, compared to the case without the optimization treatment, as illustrated in Supplementary [Supplementary-material supplementary-material-1].

To get clearer understanding of the effects of noise level and number of receivers on the system performance, we present the number of available digital states as functions of both noise level and number of receivers in Figures 3(c) and 3(d), which indicate the cases before and after the optimization, respectively. We remark that the number of controllable columns is kept the same as that in Figures 3(a) and 3(b). Obviously, as we increase the number of receivers, the numbers of available digital states with and without the optimization increase effectively. The lower the noise level is, the fewer receivers are required to reach the saturation condition. For the case before the optimization (Figure 3(c)), the number of available digital states is quite limited (see the dark purple part) if there are not many receivers (<20). The transition region between the saturation condition and the worst condition is very small, indicating that the system is sensitive to both noise level and number of receivers. However, the number of available digital states is significantly improved after the optimization (Figure 3(d)), which is confirmed by comparing Figures 3(c) and 3(d). Part of the dark purple region in Figure 3(c) becomes red in Figure 3(d), demonstrating the high efficiency of the channel optimization algorithm in expanding the transition region from the saturation condition to the worst condition and thus reducing the system sensitivity to noise. Please refer to Supplementary [Supplementary-material supplementary-material-1] for clearer view of the enhancement.

The proposed DDM wireless communication system is quite different from the MIMO system. MIMO was proposed to improve the channel capacity by using multiple transmitting antennas and multiple receiving antennas to exploit the multipath propagation [20]. However, the DDM system does not require multiple transmitters and multiple propagation paths to transmit independent information streams. MIMO requires multiple transmitters because the spatial multiplexing can only be maximized by transmitting multiple lower-rate streams from different transmitters, in which the digital information is still modulated on the amplitude, frequency and phase of the carrier waves. The typical distance between receivers for MIMO system is around half or one free-space wavelength, which is sufficiently large to reduce the signal correlation. In the DDM system, the distance between receivers is much larger since the digital information is directly modulated on the far-field radiation pattern. The significant difference between DDM and MIMO is the system architecture. In the DDM system, the digital information is directly amplified to control the digital states of each independently controllable column (or each digital unit for the 2D configuration). The complex modulation modules in the MIMO system (e.g., DAC, DUC, and mixer) are no longer required by the DDM system, leading to much simplified system architecture.

We remark that the DDM system can still work when there are objects between the transmitter and the receivers. It is obvious that any objects between the transmitter and the receivers will affect the shape of radiation pattern, with the degree of distortion depending on the distance between the obstacles and the transmitter. When the radiation pattern does experience certain deformation due to blockage, the channel optimization algorithm will select available digital states from all possible hardware codes to ensure error-free digital transmission under a given SNR, thus making the proposed DDM system adaptive to environment.

It is necessary to add a few remarks on how to increase the transmission rate of the DDM system. The bandwidth required by the system is very narrow because the radiation pattern is based on frequency domain. Therefore, the transmission rate can be easily doubled through frequency multiplexing by employing recently proposed frequency-dependent dual-functional programmable metamaterial [25], in which each coding particle possesses independent digital states at two distinct frequencies. Further extending the method to multi-band programmable metamaterials could enable even higher transmission rate. The other approach to increase the transmission rate without occupying extra physical bandwidth is to employ higher-order modulation, which is one of the advantages of the DDM system over the conventional communication system. For the satellite ground communication, SNR is quite limited due to the limited power supply on the satellite. To keep the system BER under a certain value, the maximum modulation order is typically chosen as 4 using the QPSK modulation [1]. For the DDM-based satellite ground communication, however, it is much easier to adopt high-order modulations because the radiation pattern at hundreds of kilometers away can be easily sampled with high spatial resolutions, i.e., with more ground stations.

Another distinctive characteristic of the DDM system indicates that the transmitted information is distributed in different directions of the radiation pattern, making it possible for perfectly secret communication. Firstly, it is practically impossible to intercept the radiation pattern at different angles simultaneously, especially for the satellite ground communication where the neighboring receivers are commonly hundreds of miles apart. Secondly, even if we assume that the eavesdroppers have the ability to get the entire radiation pattern, they are still unable to recover the original digital information without having the knowledge of channel condition. For example, which receivers are currently under use? How to map the available digital states to the original information? This is similar to frequency-hopping (FH) technique used in the modern communication systems for secrete communication [3], where the signals rapidly change among a pre-determined order of sub-frequencies (a pseudorandom sequence) known to both transmitter and receiver. For the DDM system, the channel condition is dynamically evaluated for better system performance and safer data transmission. In this regard, dynamically changing digital states can be compared to the pseudorandom sequence in the FH technique. But unlike the FH technique which needs much wider bandwidth, the DDM-based secret communication does not require extra bandwidth.

**Figure 4:**
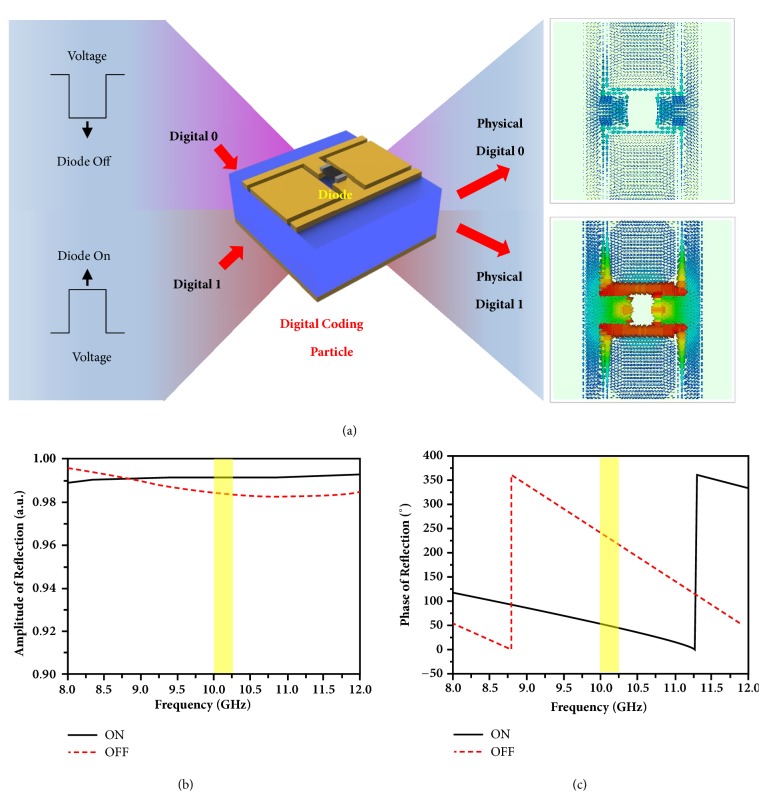
**Design of the digital coding particle for programmable metasurface and its reflection responses at “ON” and “OFF” states.** (a) The designed digital coding particle and its induced surface currents when the diode is at “ON” and “OFF” states, which builds up a bridge between the digital world and physical world. (b, c) The reflection amplitude (b) and phase (c) responses of the digital coding particle when the diode is at “ON” and “OFF” states.

For experimental validation, we firstly realize the programmable metamaterial using a specially designed digital coding particle, as illustrated in Figure 4(a) and Supplementary [Supplementary-material supplementary-material-1]. The coding particle is composed of a resonant metallic pattern and a pin-diode (Skyworks, SMV-1230), which are located on a metal-backed dielectric film with the thickness of 3mm and relative permittivity of 2.65+*i∗*0.003. Figure 4(a) clearly shows how the coding particle reacts to EM waves under different digital states of information codes (‘0' state: zero voltage, the diode ‘OFF'; ‘1' state: 3.3V voltage, the diode ‘ON'), producing different EM responses (induced surface currents) with physical digital coding states ‘0' and ‘1'. Here, the diode is modelled as a lumped circuit element with series resistor-inductor-capacitor (RLC) circuit parameters R=0.5Ω, L=0.75nH, and C=0pF for the ‘ON' state and R=0Ω, L=0.5nH, and C=0.4pF for the ‘OFF' state. The reflection magnitude and phase responses are presented in Figures 4(b) and 4(c), respectively. We observe that a 180° phase difference is obtained in the operational frequency 10.15GHz with the amplitude of reflection over 0.98.

### 2.3. Experimental Realization of the DDM Prototype

**Figure 5:**
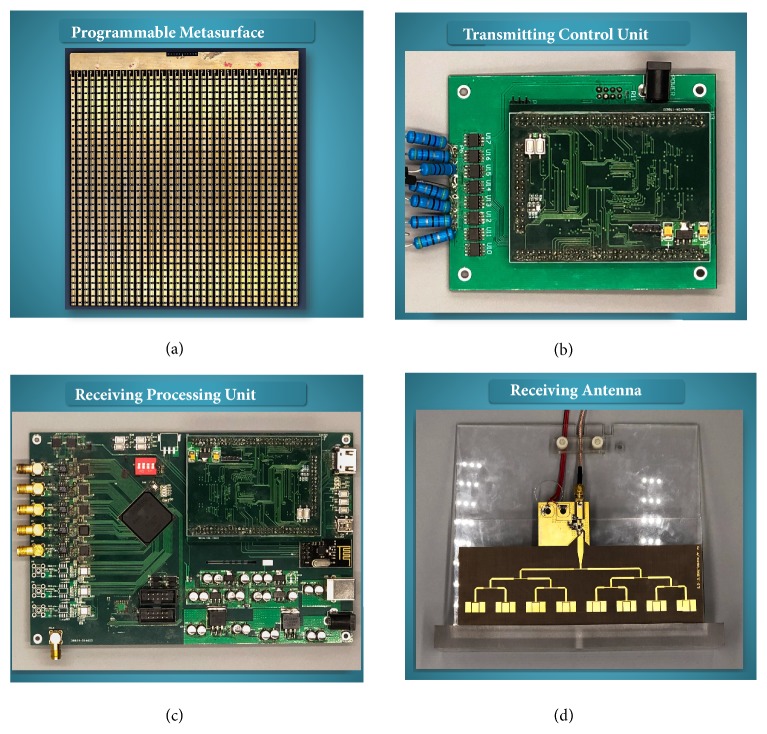
**Photographs of the fabricated programmable metasurface.** (a) Transmitting control unit (b) receiving processing unit (c) and receiving antenna (d) respectively, for the DDM wireless communication system.

We then fabricate and characterize a prototype of DDM communication system, which is composed of a programmable metasurface (Figure 5(a)), a transmitting control unit (Figure 5(b)), a receiving processing unit (Figure 5(c)), and two receiving antennas (Figure 5(d)). The system setup is demonstrated in Supplementary [Supplementary-material supplementary-material-1]. The programmable metasurface, comprising of an array of 35×35 digital particles, is dynamically controlled by the transmitting control unit. Under the illumination of feeding antenna, which connects to a PSG vector signal generator (Agilent E8267D OPT H44) with 25-dBm power at 10GHz, the programmable metasurface with 7 independently controllable columns (5 columns construct one controllable column) radiates a total number of 2^6^=64 different radiation patterns to the free space, in which the first controllable column is constantly set as “0” state. The performance of the fabricated programmable metasurface was experimentally confirmed in an anechoic chamber (see Supplementary [Supplementary-material supplementary-material-1]). Good match between the simulated and measured radiation patterns is observed from Supplementary [Supplementary-material supplementary-material-1]. The receiving processing unit samples the voltages sent from the two receiving antennas at a rate of 5MHz, converts them into digital data, and processes them in MCU to recover the original digital information according to a pre-stored look-up table, which is established in the channel auto-scan step using the channel estimation algorithm and channel optimization algorithm. The method section provides detailed information for each module. The complete workflow of the DDM prototype is sketched in Supplementary [Supplementary-material supplementary-material-1] and is briefly described in the method section.

**Figure 6:**
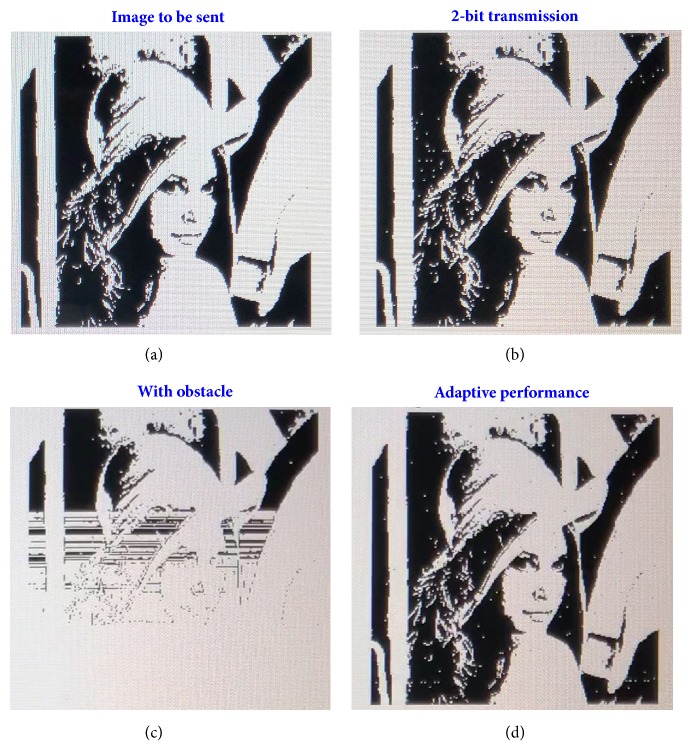
**Measurement results of the DDM wireless communication system.** (a) The original image to be transmitted. (b) The received image under the 2-bit-symbol transmission mode. (c) The received image when a metal plate is placed between the programmable metasurface and two receiving antennas. (d) The received image after running the channel autoscan program when a metal plate is placed between the programmable metasurface and the two receiving antennas.

As example, we transmit a binary image with 7.2k bytes (see Figure 6(a)) to demonstrate the performance of the DDM prototype. In the first scenario, two receiving antennas are placed at 18° and 28° with respect to the metasurface normal at a distance of 50cm, respectively. By performing the auto-scan process, we obtain five available digital states, which are Nos. 41, 47, 51, 61, and 63, from which the first four digital states are selected to support the 2-bit-symbol transmission mode. It takes about 58s to transmit the entire image, equivalent to an average transmission speed of 124 Byte/s. The received image is illustrated in Figure 6(b), from which we could see that the image is correctly recovered with high fidelity compared to the original image (Figure 6(a)). Supplementary [Supplementary-material supplementary-material-1] records the entire transmitting process.

In the second scenario, a metal plate was placed between the programmable metasurface and two receiving antennas to mimic the obstacle condition, which is commonly encountered in practical applications. Transmission error occurs as soon as we insert the metal plate, as shown in Figure 6(c) and Supplementary [Supplementary-material supplementary-material-1], because the radiation pattern is perturbed and the look-up table is no longer valid. To demonstrate the adaptive performance of the DDM prototype and correctness of the proposed channel optimization algorithm, in the third testing scenario, we re-execute the channel auto-scan program with the existence of obstacle. In this case, we are able to find four digital states (Nos. 45, 51, 61, and 63), in which three digital states (numbers 45, 51, and 61) are selected to support 1-bit-symbol transmission, while the digital state 63 is used for the state flag. The received image is demonstrated in Figure 6(d), and the transmission process is given in Supplementary [Supplementary-material supplementary-material-1]. We clearly see that the image quality is as good as that in the first scenario.

We remark that several factors restrict the transmission distance of the DDM prototype, for example, only two receivers, the limited sensitivity of the demodulating amplifier chip at 10GHz, the circuit noise of the receiving circuit board, the limited transmitting power allowed by the RF source, and lack of error correction algorithm. The transmission distance can be extended effectively by improving SNR at the receiving side by using high-sensitivity demodulating amplifier chip, high gain antenna, and anti-jamming technique. Due to the instability of the temporarily designed receiving processing unit, the symbol rate is set at a relatively low level of 496 symbols per second to guarantee the error-free transmission. As the transmitting speed depends on both symbol rate and modulation order, which are determined by the forward recover time of diode (much faster than the reverse recovery time of 5~10ns) and the number of controllable columns, respectively, a faster transmission speed of over 100 Mbps could be expected by considering the above engineering issues.

## 3. Discussion

The proposed DDM architecture based on the programmable metasurface opens a plethora of opportunities for designing new wireless communication systems with outstanding features such as high spectral efficiency and much simplified system architecture. The most unique characteristic is the radiation pattern modulation technique, in which the information is distributed in a wide range of angles in the far fields, making an eavesdropper impossible to intercept the transmission message at any single or multiple positions. Based on the recent finding of space-time coding digital metasurface, we remark that the information of the DDM system can be modulated not only in the spatial domain, but also in the frequency domain, making the system more efficient in utilizing the physical bandwidth [26–28]. Despite the fact that we only present in this work the simplified communication mode in the first prototype, we are currently seeking to allow it to work under duplex operation mode by letting each transmitter as receiver and all transceivers being synchronized wirelessly. In addition, real-time channel estimation and optimization can be readily realized, enabling the system to be dynamically adjusted for real-time transmissions under any circumstances. Borrowing some prevalent techniques in the traditional wireless communication systems, the propose DDM architecture is expected to have more features and better performance, and is likely to revolutionize the wireless communication industry, especially in the field of security communication.

## 4. Materials and Methods

### 4.1. DDM System Setup

*Transmitter. *The transmitter is composed of a programmable metamaterial, a transmitting control unit, and a feeding antenna. The transmitting control unit mainly comprises of a microcontroller unit (MCU, ST, STM32F767IGT6) and an FPGA (Altera, EP4CE15F23C8). Both cores communicate through the SPI (serial peripheral interface) protocol. The MCU is responsible for interaction between the transmitter and receiver through a wireless transceiver module (NRF24L01, Nordic, nRF24L01). FPGA is controlled under the instructions of MCU, and adjusts the digital states of each controllable column on the programmable metasurface. The feeding antenna, working from 9.84 to 15 GHz with a gain of 20 dBi, illuminates the programmable metasurface at an angle of 20°.

*Receiver. *The receiver comprises of a receiving processing unit and two receiving antennas. The receiving processing unit is also composed of an MCU (STM32) and an FPGA (Altera, EP4CE6E22C8). MCU controls the communication between the transmitter and receiver, the implementation of channel estimation/optimization algorithms, and human-computer interaction, while FPGA records the multiple channels of digital signals output from ADC (ADI, AD9629). Note that the receiving processing unit supports a maximum number of 8 channels.

*Receiving Antenna. *The receiving antenna is made of a microstrip antenna array and a demodulating logarithmic amplifier chip (ANALOG DEVICES, AD8317). To match working bandwidth of the programmable metasurface, the microstrip antenna array is designed to work from 9.2 to 10.5GHz with gain of 16.4dBi and half-power bandwidth (HPBW) of 7°. In the experiment, the microstrip antenna should aim at the programmable metasurface to maximize the intensity of the received RF signal. The demodulating logarithmic amplifier chip, with a -28dBm sensitivity at 10GHz, is employed to convert the received RF signal to a DC voltage ranging from 0.8V to 1.5V in a decibel scaling, which indicates the intensity of the scattered EM wave at each receiving position.

### 4.2. Workflow of the DDM Prototype

**Step 1 (channel auto-scan).**

To obtain the available digital states that can be used in the current system configuration and environment (e.g., the number of receiving antennas, their positions, and the wireless channel condition), the transmitting control unit sends 64 digital states from 0 to 63 by biasing the corresponding voltages on the programmable metasurface. To minimize the inaccuracy caused by the environment noise, circuit noise, and any inevitable errors, each digital state is sampled with a period of 150ms to allow the receiving processing unit to get an average result from 16 samplings. The digital signals collected from multiple receivers are stored at the receiving MCU, and processed by a built-in channel optimization algorithm to generate all available digital states that could be used for a reliable transmission at a given noise threshold. Then the receiving MCU determines 2^*n*^ digital states from the available digital states and notifies the transmitting MCU through the wireless transceiver module. A look-up table mapping the relation between n-bit long symbols and selected digital states is established and is sent to the transmitting MCU. Now, the system is ready for data transmission at* n* bits per symbol rate.

**Step 2 (data sending).**

The transmitting MCU splits the data to be sent (e.g., an image in our demo) into multiple* n*-bit-long symbols, then interprets them into the hardware codes based on the look-up table, and finally transmits them into free space though the programmable metasurface.

**Step 3 (data receiving).**

In each symbol period, the receiving MCU receives the digital data from the multiple channels, and recovers the original data based on the look-up table. The complete data (e.g., images) can be received and displayed on the screen by repeating Steps 1 and 2 at a certain symbol rate.

## Supplementary Materials

Supplementary Materials Figure S1: Schematic illustrations of traditional wireless communication system and popular modulation techniques. Figure S2: The 16 digital coding patterns of a 5-column DDM wireless communication system. Figure S3: Radiation patterns in the* x-z* plane of the 16 digital coding patterns. Figure S4: Enhancement of the number of available digital states by the channel optimization algorithm. Figure S5: Detailed design parameters of the digital coding particle of the programmable metasurface. Supplementary Figure S6: Photograph of the DDM wireless communication system prototype. Figure S7: Experimental characterization of the radiation pattern for the fabricated programmable metasurface in an anechoic chamber. Figure S8: Simulated and measured radiation patterns for four different coding sequences. Figure S9: The work flow of the DDM wireless communication system. Figure S10: Working-mechanism demonstration of the channel optimization algorithm by showing the locations of the 16 digital states of a 5-column DDM system in a 2D Cartesian coordinate system. Movie 1: Transmission testing of DDM system with 2-bit-symbol transmission mode. Movie 2: Transmission with an obstacle. Movie 3: Adaptive performance of the DDM prototype.
