# Supplementary material for: Direct Transmission of Digital Message via Programmable Coding Metasurface
Source: Research (Wash D C). 2019 Jan 16;2019:2584509. doi: 10.34133/2019/2584509 (PMC6750087; doi:10.34133/2019/2584509)
Supplement: Supplementary Materials — Figure S1: Schematic illustrations of traditional wireless communication system and popular modulation techniques. Figure S2: The 16 digital coding patterns of a 5-column DDM wireless communication system. Figure S3: Radiation patterns in the x-z plane of the 16 digital coding patterns. Figure S4: Enhancement of the number of available digital states by the channel optimization algorithm. Figure S5: Detailed design parameters of the digital coding particle of the programmable metasurface. Supplementary Figure S6: Photograph of the DDM wireless communication system prototype. Figure S7: Experimental characterization of the radiation pattern for the fabricated programmable metasurface in an anechoic chamber. Figure S8: Simulated and measured radiation patterns for four different coding sequences. Figure S9: The work flow of the DDM wireless communication system. Figure S10: Working-mechanism demonstration of the channel optimization algorithm by showing the locations of the 16 digital states of a 5-column DDM system in a 2D Cartesian coordinate system. Movie 1: Transmission testing of DDM system with 2-bit-symbol transmission mode. Movie 2: Transmission with an obstacle. Movie 3: Adaptive performance of the DDM prototype. [file 2584509.f1.zip › 2584509.f1/SM_Research_20180910.docx]

**Supplementary Material for**

**Direct transmission of digital message via programmable coding metasurface**

Tie Jun Cui^1,2,3,†,*^, Shuo Liu^1,2,3,†^, Guo Dong Bai^1,2,†^, and Qian Ma^1,2^

^1^ State Key Laboratory of Millimeter Waves, Southeast University, Nanjing 210096, China

^2^ Synergetic Innovation Center of Wireless Communication Technology, Southeast University, Nanjing 210096, China

^3^ Jiangsu Xuantu Technology Co., Ltd., 12 Mozhou East Road, Nanjing 211111, China

† These authors contributed equally to this work.

* Corresponding author: tjcui@seu.edu.cn

**This PDF file includes:**

Supplementary Text

Supplementary Figure S1 to S10

Supplementary Videos 1-3

Supplementary Reference

**Supplementary Text**

**Experimental Details**

We take the simple case of a 5-column DDM system with two sampling angles as example to interpret the channel optimization algorithm in a more intuitive way. Because each digital state is described by two variables, it can be plotted in a 2D Cartesian coordinate system. Supplementary Figure S10a shows the locations of 16 digital states in the 2D plot when the radiation pattern is sampled at 10° and 30°. As is mentioned in the main text, the digital states 16, 9, 8, 12, 13 and 14 have the same locations with digital states 2, 3, 4, 6, 7 and 10, respectively. If we set a noise threshold value G to the system, all digital states with mutual distance smaller than G will be considered as a single digital state. As a result, there will be only one available digital state when the noise threshold is set as 0.3, resulting in the worst condition that no information can be transmitted. If we draw two circles with radius 0.3 centered at the locations of digital states 1 and 5, as illustrated by the two red circles in Supplementary Figure S10a, we should notice that the digital states 3/9 fall into both circles, which makes digital states 1 and 5 connected. Because the distance between digital states 1 and 5 is larger than 0.3, they can be separated as two distinct digital states if digital states 3/9 are deleted from the 16 digital states. Now the system will be able to support 1 bit/symbol transmission if digital states 1 and 5 are selected as two available digital states. Clearly, this is not the only solution. There are some other selections such as 2/16 and 11, 4/8 and 10/14, 5 and 4/8, etc., which also support 1 bit/symbol transmission. However, at the current noise level, the maximum number of available digital states can only be optimized as 2. There are two possible ways to further increase the number of available digital states. One is to sample the radiation pattern at other angles, or to increase the number of sampling. The other way is to lower the noise level, which is equivalent to increase SNR of the system.

In the second case, we change the sampling angle to 20° and 40°, and the resulting locations of the 16 digital states are plotted in Supplementary Figure S10b. Similar to the first case, only one available state can be found by performing the channel estimation algorithm to the distance matrix when the noise threshold is set as 0.3. However, it is exciting to find that new locations of the 16 digital states bring more available digital states after performing the channel optimization algorithm. The four circles with radius 0.3 show that, if we delete the digital states 2, 4/8, 5, we could obtain four available digital states: 1, 3/9, 6/12, and 7/13.

For the third case, we reduce the noise threshold to 0.1 while keeping the sampling angle the same as the first case. Supplementary Figure S10c shows that the total number of available digital states increase to 9, which are 1, 2/16, 3/9, 4/8, 5, 6/12, 7/13, 10/14, 11. However, because no digital state links two or multiple states, the optimization algorithm cannot provide any additional digital states.

We should note that although the working mechanism of the channel optimization algorithm is demonstrated here with the 2D case, it also applies to the complicated cases where the radiation pattern is sampled at multiple angles, in which the search of the maximum number of digital states is conducted in higher dimensional space.


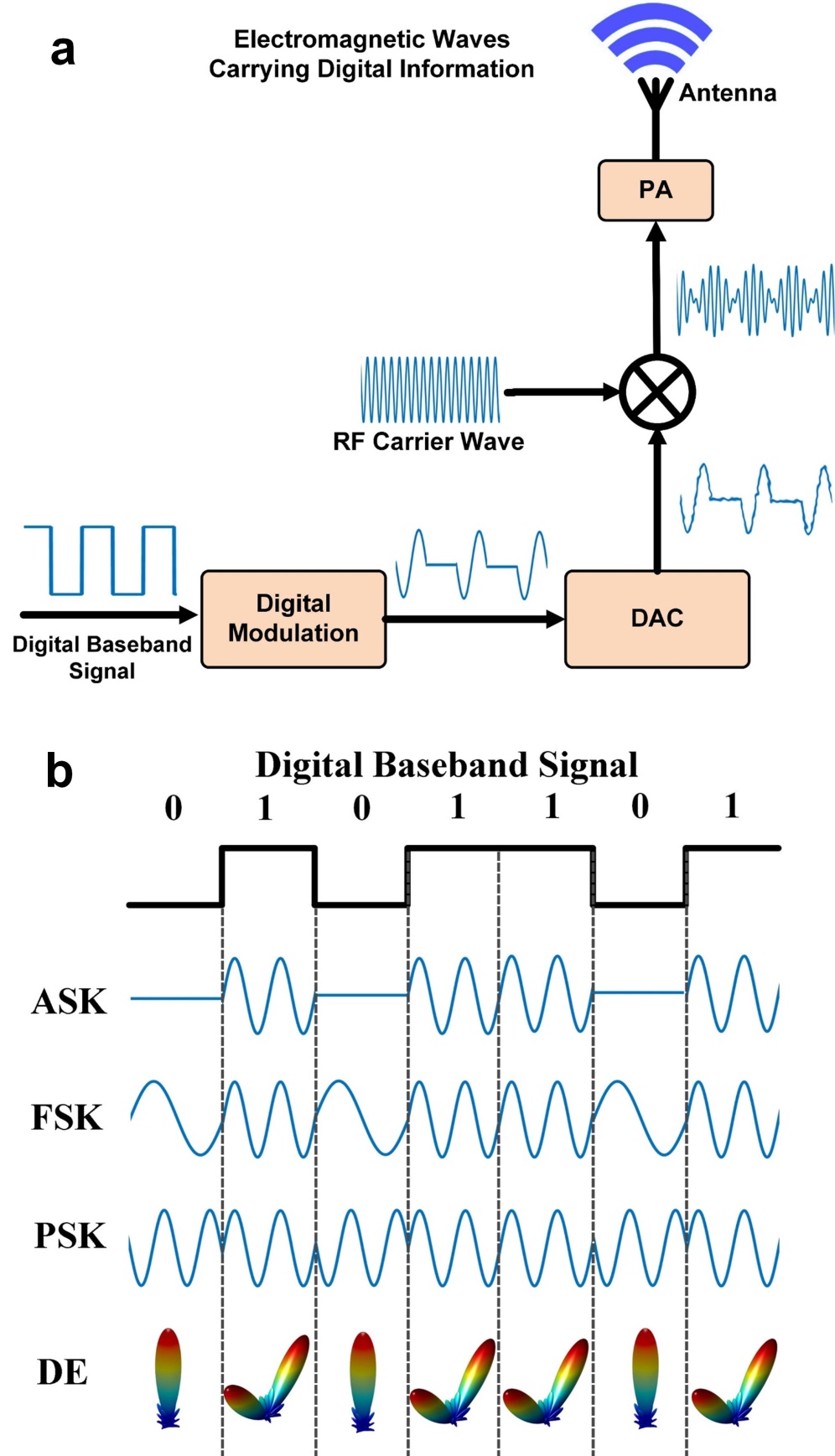


**Supplementary Figure S1. Schematic illustrations of traditional wireless communication system and popular modulation techniques. (a)** Schematic of the traditional wireless communication system. **(b)** The mostly used modulation techniques ASK, FSK, and PSK for traditional wireless communication systems and the radiation-pattern-based modulation for the proposed DDM system.


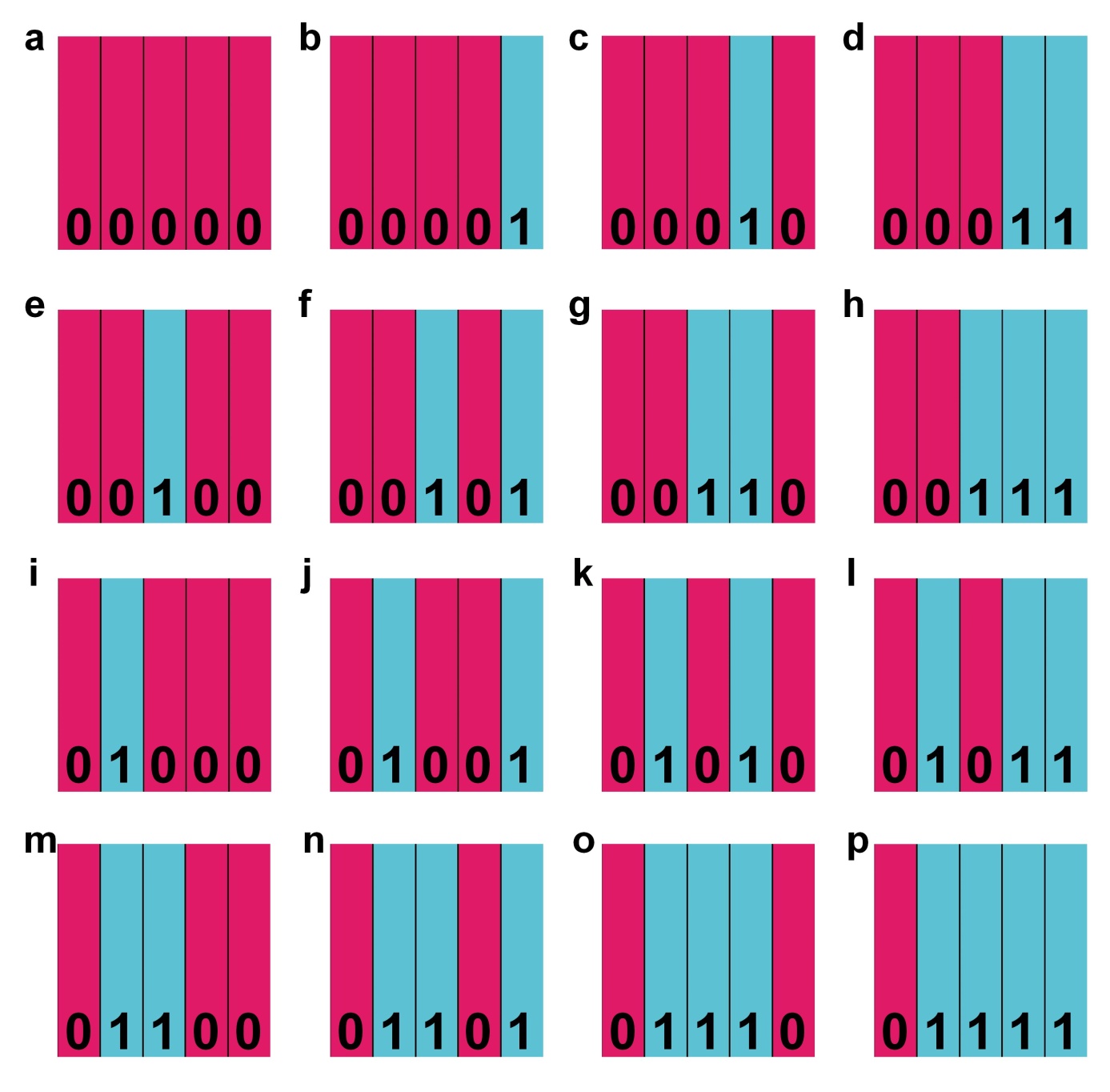


**Supplementary Figure S2. The 16 digital coding patterns of a 5-column DDM wireless communication system.**


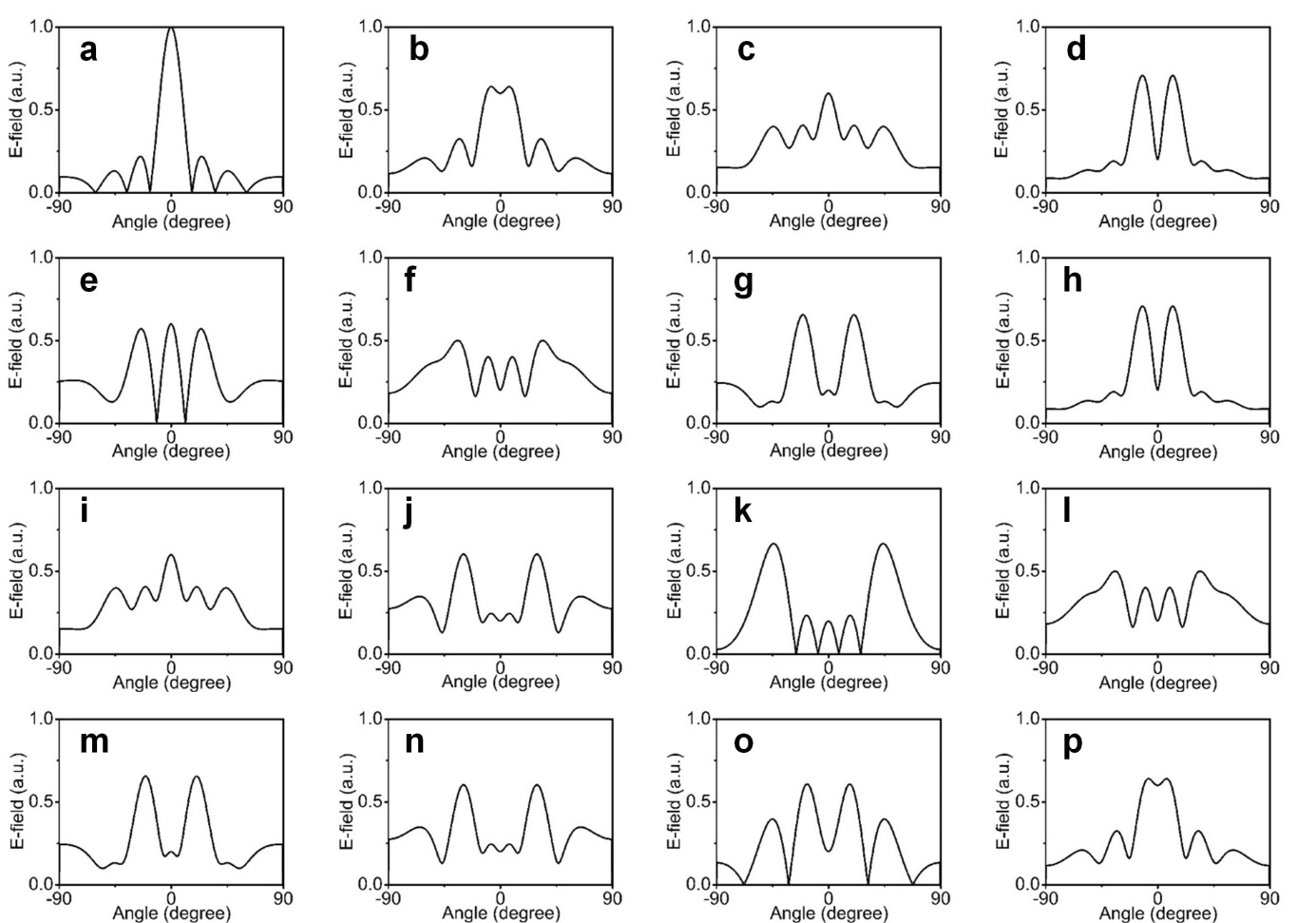


**Supplementary Figure S3. Radiation patterns in the *x-z* plane of the 16 digital coding patterns.**


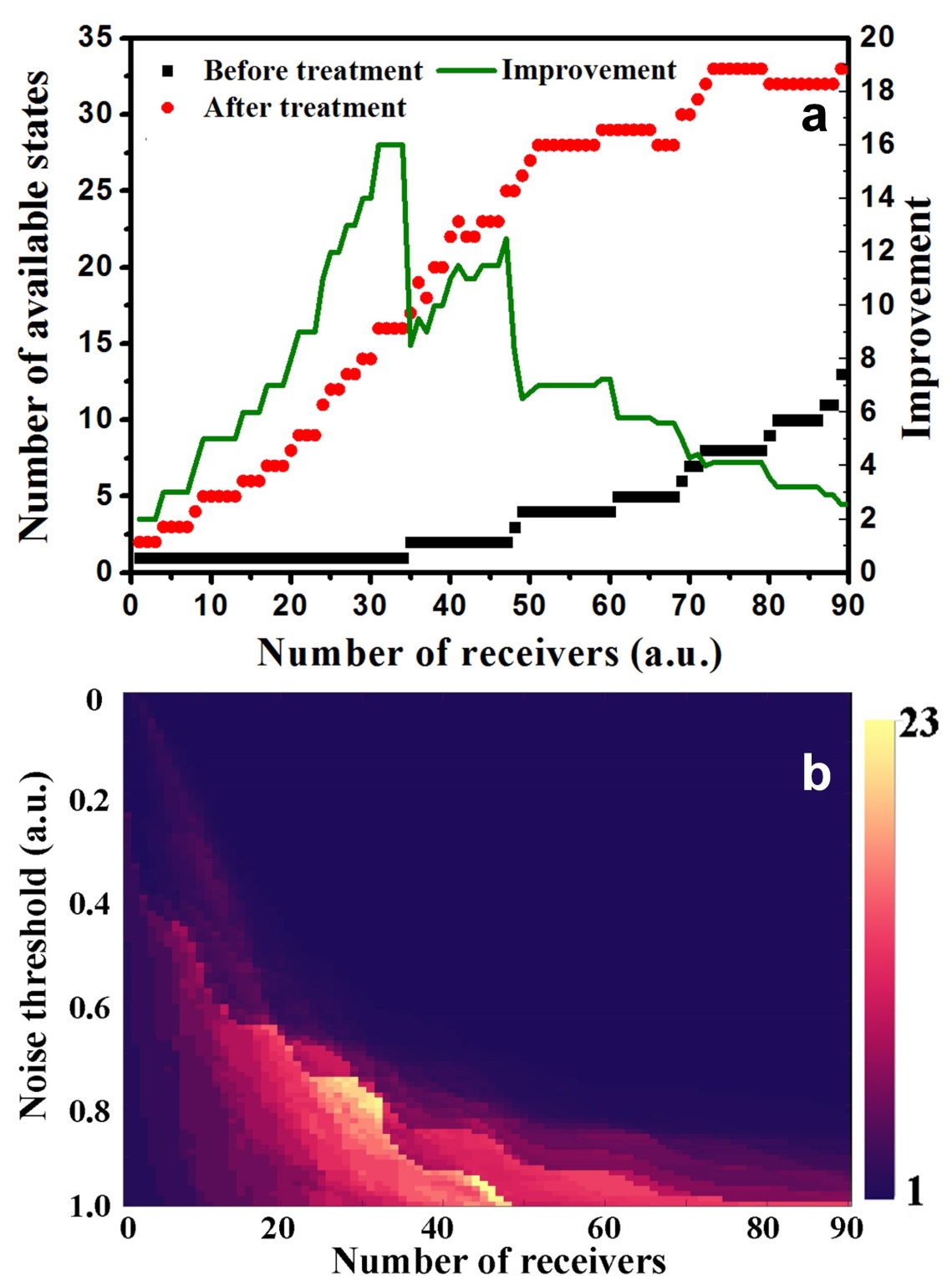


**Supplementary Figure S4. Enhancement of the number of available digital states by the channel optimization algorithm. (a)** The number of available digital states before and after the optimization treatment for the case in Figure 3b with the noise threshold 0.9. The green line indicates the enhancement factor of the number of available digital states after the optimization treatment. **(b)** The enhancement factor of the available digital states enabled by the channel optimization algorithm. It is obtained by dividing Figure 3d by Figure 3c. The bright region indicates the range of condition where the optimization algorithm can provide the maximum improvement.


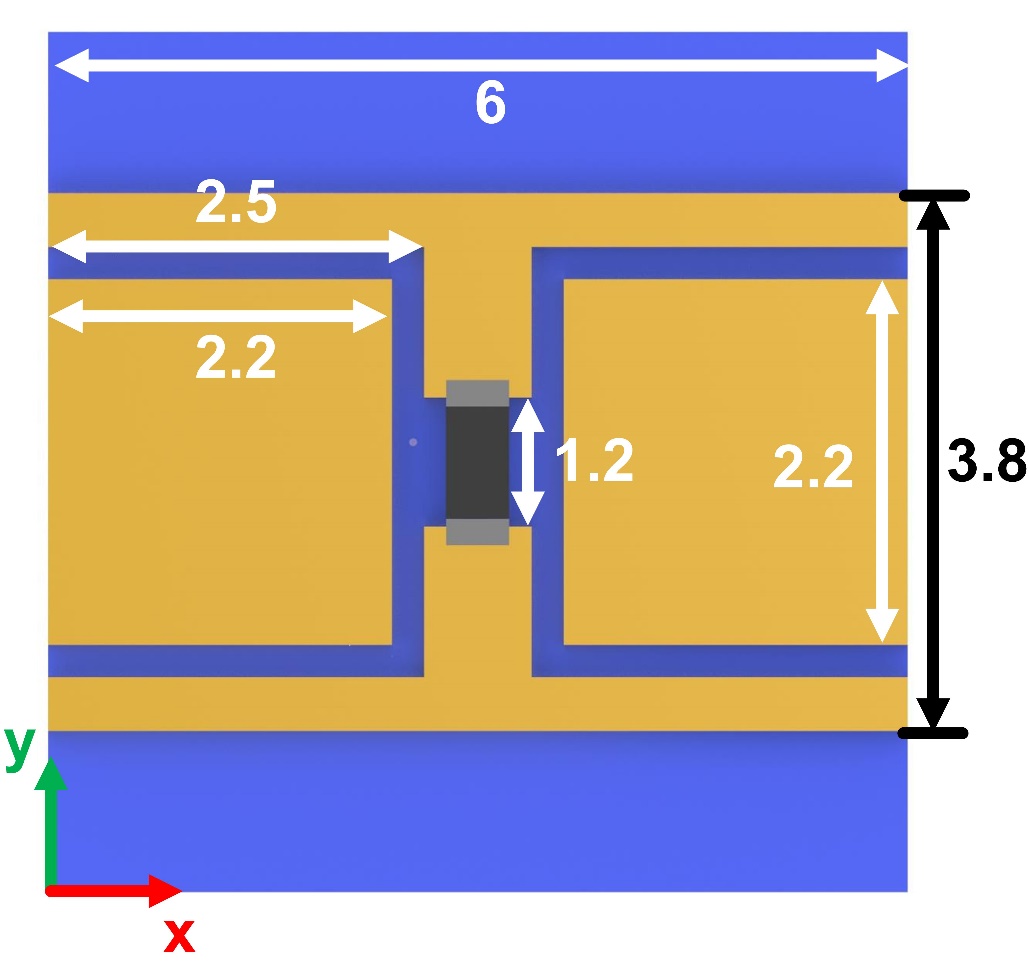


**Supplementary Figure S5. Detailed design parameters of the digital coding particle of the programmable metasurface**.


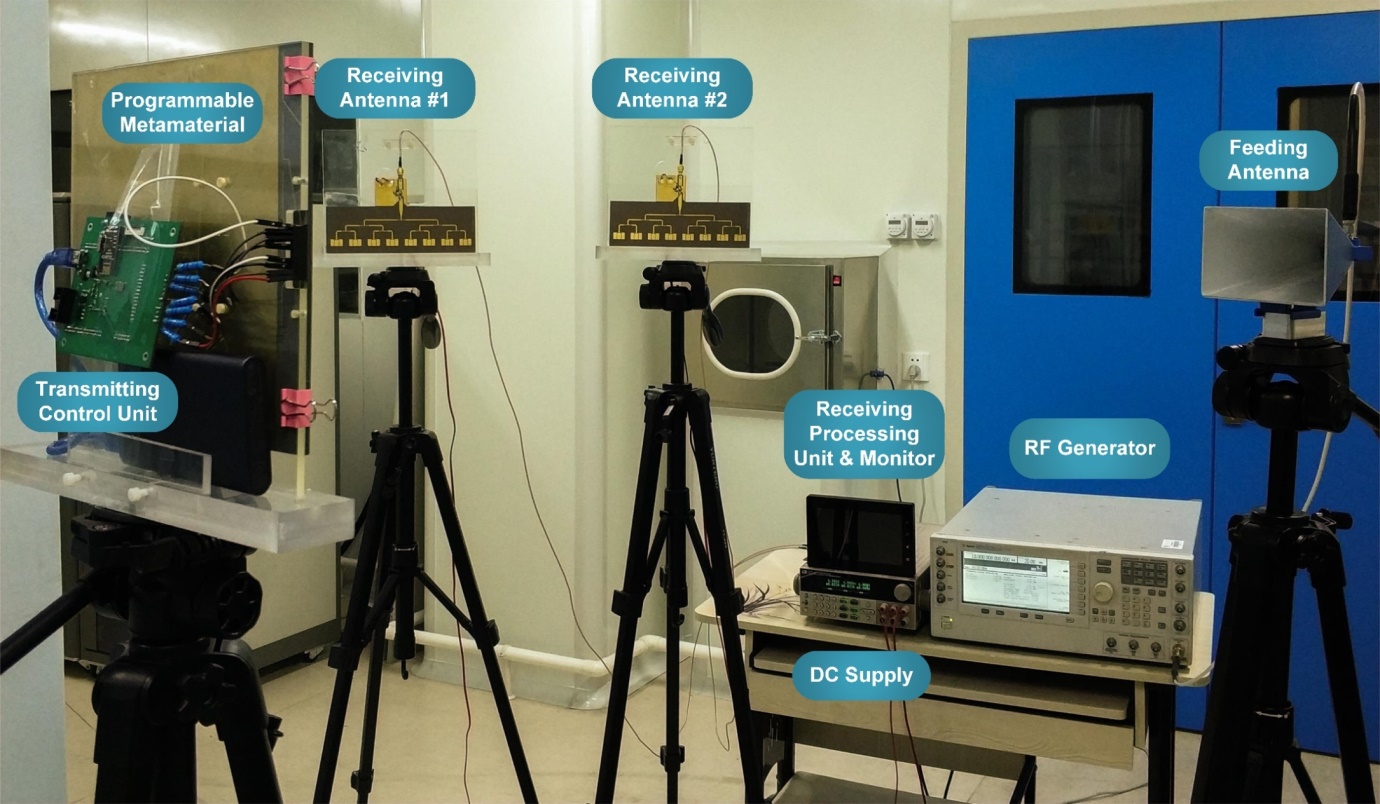


**Supplementary Figure S6. Photograph of the DDM wireless communication system prototype**.


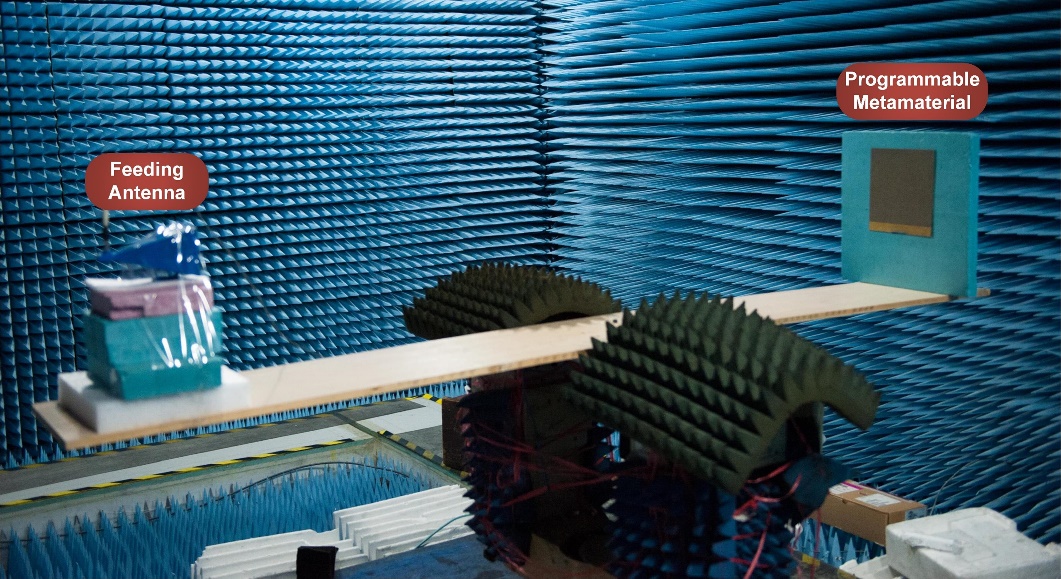


**Supplementary Figure S7. Experimental characterization of the radiation pattern for the fabricated programmable metasurface in an anechoic chamber. The programmable metasurface and horn antenna are placed at both ends of a long wooden board, which could automatically rotate 360° to record the far-field radiation fields by the receiving antenna. The horn antenna and programmable metasurface are kept at a distance of 1.8m to eliminate the phase difference on the programmable metasurface. Please refer to Ref. S1 for more details of the experimental configuration.**


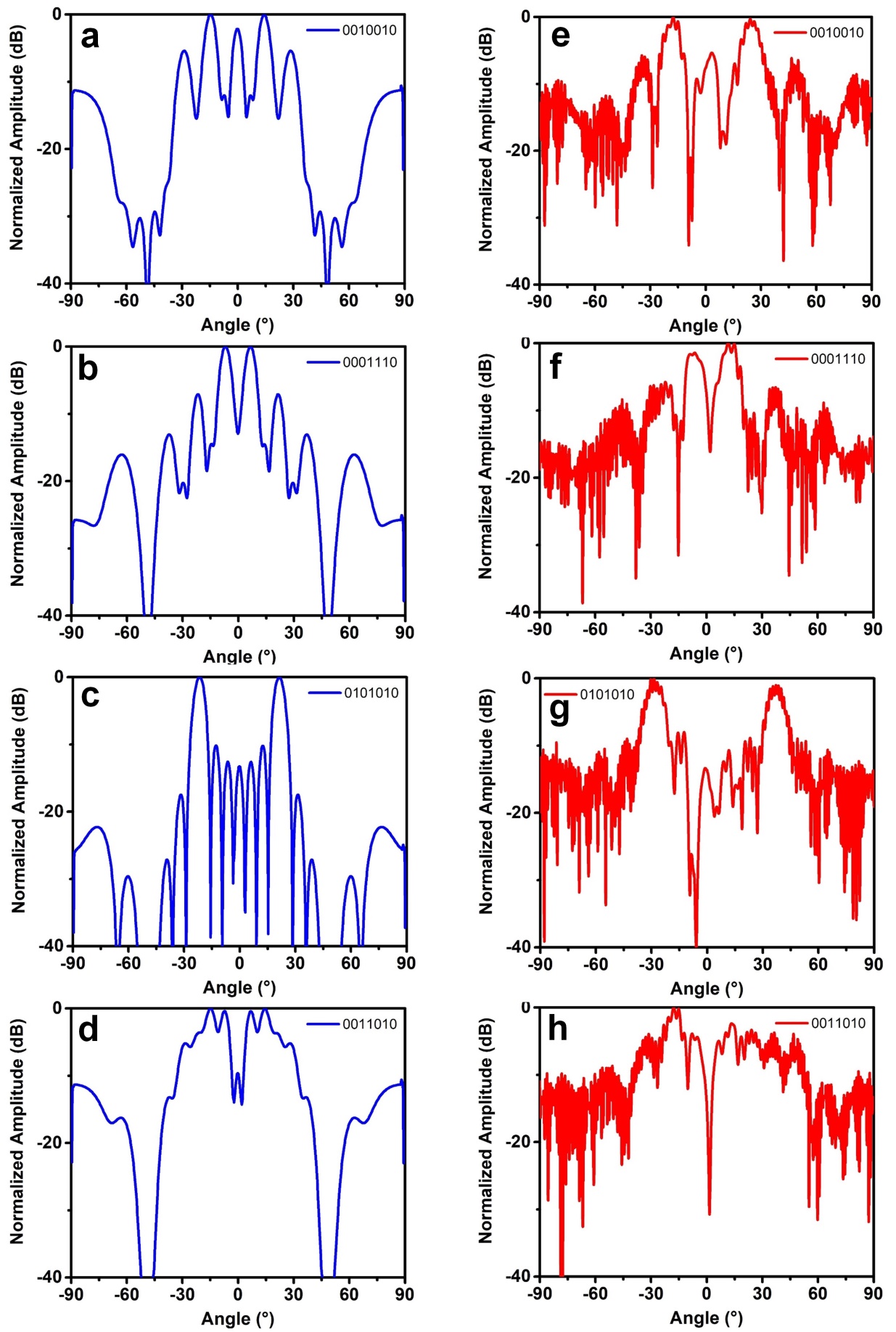


**Supplementary Figure S8. Simulated and measured radiation patterns for four different coding sequences. (a-d)** Simulated radiation patterns for the coding sequences 0010010, 0001110, 0101010, and 0011010, respectively. **(e-h)** Measured radiation patterns for coding sequences 0010010, 0001110, 0101010, and 0011010, respectively.


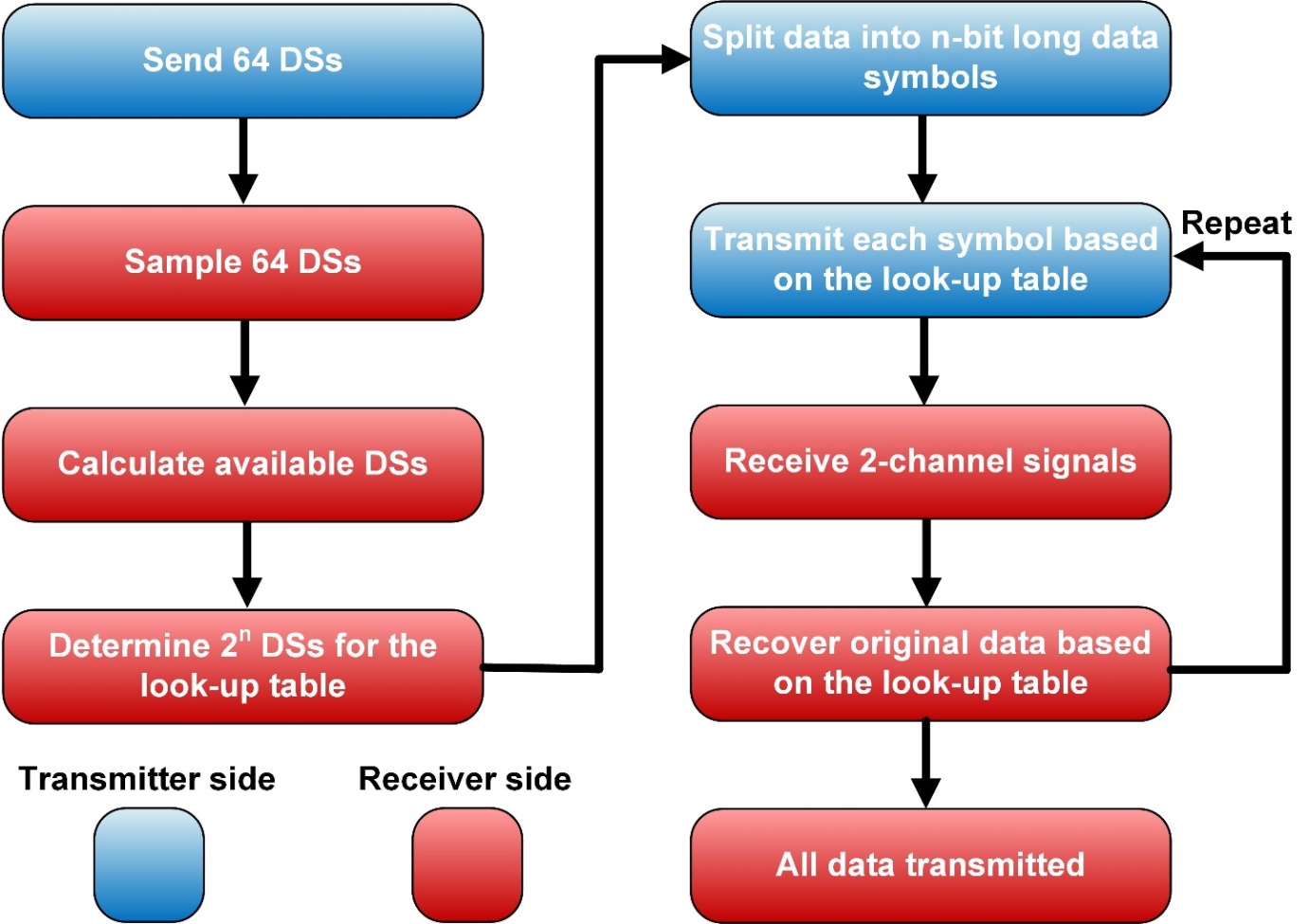


**Supplementary Figure S9. The work flow of the DDM wireless communication system.**


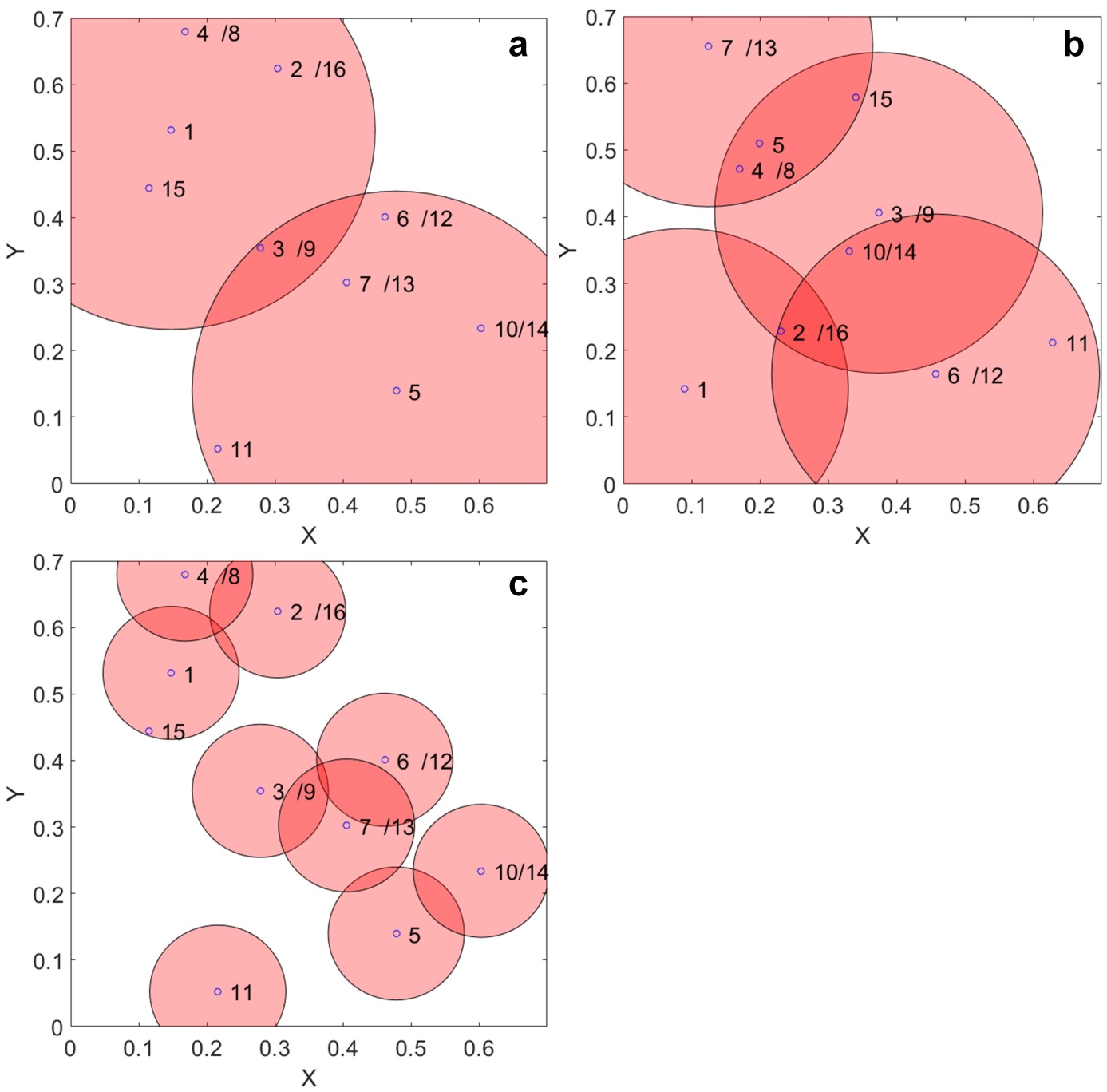


**Supplementary Figure S10. Working-mechanism demonstration of the channel optimization algorithm by showing the locations of the 16 digital states of a 5-column DDM system in a 2D Cartesian coordinate system. The centers of circles in all plots are the available digital states selected by the optimization treatment. (a)** When the radiation pattern is sampled at 10° and 30° and the noise threshold is set as 0.3. **(b)** When the radiation pattern is sampled at 20° and 40° and the noise threshold is set as 0.3. **(c)** When the radiation pattern is sampled at 10° and 30° and the noise threshold is set as 0.1.

**Supplementary Movie 1. Transmission testing of DDM system with 2-bit-symbol transmission mode.** In the video, the image is correctly received with high fidelity.

**Supplementary Movie 2. Transmission with an obstacle.** When a metal plate inserted in the transfer route of the DDM system, transmission error occurs.

**Supplementary Movie 3. Adaptive performance of the DDM prototype.** Re-execute the channel auto-scan program, new available digital states are obtained, which can support 1-bit-symbol transmission. Even though the obstacle remained, the image can be successfully transmitted.
